# Supplementary material for: A Tough and Mildew-Proof Soybean-Based Adhesive Inspired by Mussel and Algae
Source: Polymers (Basel). 2020 Mar 31;12(4):756. doi: 10.3390/polym12040756 (PMC7240608; doi:10.3390/polym12040756)
Supplement: Supplementary file 1 [file polymers-12-00756-s001.pdf]

Supporting Information

A Tough and Mildew-proof Soybean-based Adhesive Inspired by Mussel and Algae

Yue Bai [1,2,3], Xiaorong Liu [1,2,3], Sheldon Q. Shi [2,4], Jianzhang Li [1,2,3,*]

[1] Beijing Advanced Innovation Center for Tree Breeding by Molecular Design, Beijing Forestry University, Beijing 100083, China; byueer@126.com (Y.B.); happyrong1993@bjfu.edu.cn (X.L.)
[2] Key Laboratory of Wood Materials Science and Utilization, Beijing Forestry University, Beijing 100083, China; sheldon.shi@unt.edu
[3] College of Materials Science and Technology, Beijing Forestry University, Beijing 100083, China
[4] Department of Mechanical and Energy Engineering, University of North Texas, Denton, TX 76203, USA
* Correspondence: lijzh@bjfu.edu.cn; Tel./ Fax: +86−010− 62338356

Received: 23 February 2020; Accepted: 26 March 2020; Published: date

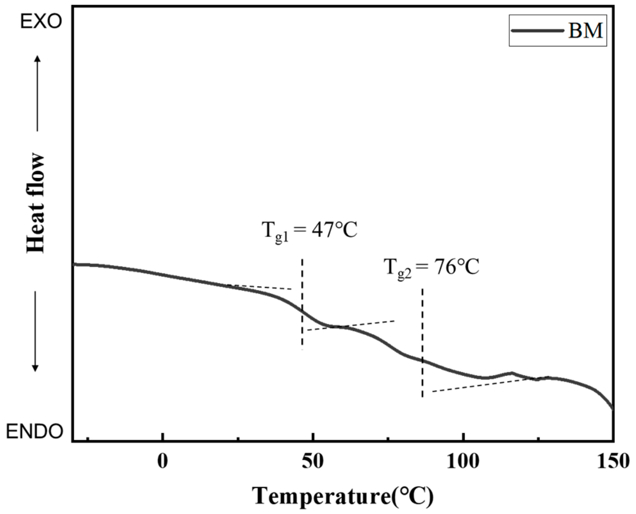

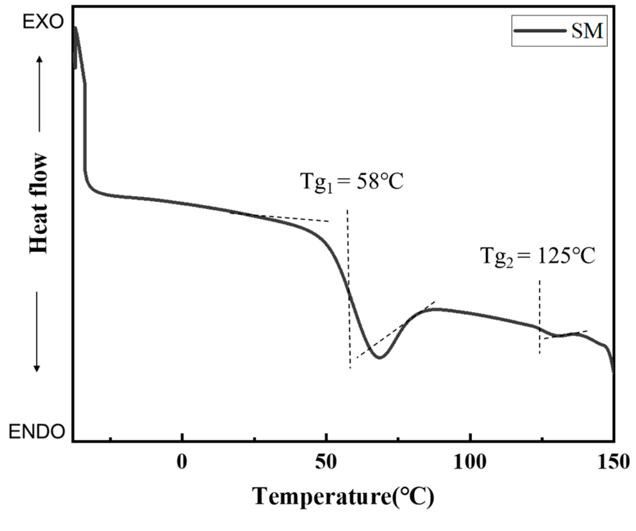

Figure S1. DSC curves of pristine soybean meal (SM) and blood meal (BM).

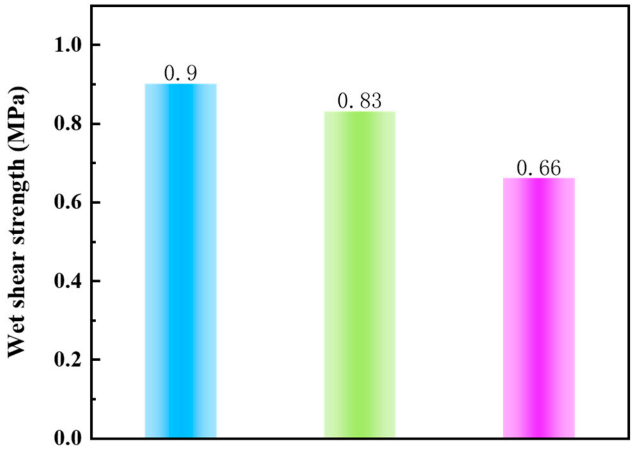

Figure S2. The wet shear strength of plywood bonded by modified SM/BM-based adhesives.

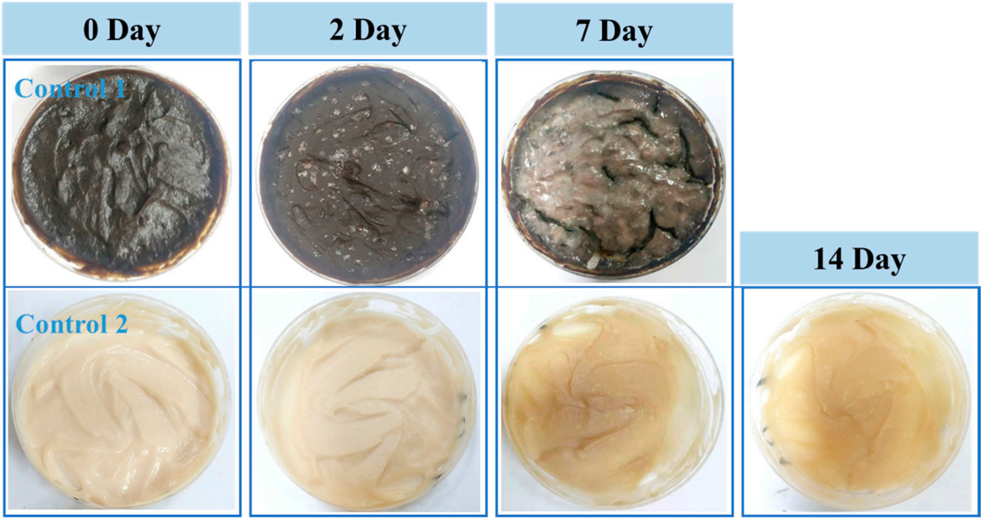

Figure S3. Mildewproof activities of the control adhesives under different mildew treatment time.
